# Impacts of antipsychotic medication prescribing practices in critically ill adult patients on health resource utilization and new psychoactive medication prescriptions

**DOI:** 10.1371/journal.pone.0287929

**Published:** 2023-06-29

**Authors:** Natalia Jaworska, Andrea Soo, Henry T. Stelfox, Lisa D. Burry, Kirsten M. Fiest

**Affiliations:** 1 Department of Critical Care Medicine, Cumming School of Medicine, University of Calgary, Calgary, AB, Canada; 2 Alberta Health Services, Calgary, AB, Canada; 3 Department of Community Health Sciences, Cumming School of Medicine, University of Calgary, Calgary, AB, Canada; 4 Leslie Dan Faculty of Pharmacy, O’Brien Institute for Public Health, Cumming School of Medicine, University of Calgary, Calgary, AB, Canada; 5 Departments of Pharmacy and Medicine, Mount Sinai Hospital, University of Toronto, Toronto, Canada; 6 Department of Psychiatry, Cumming School of Medicine, University of Calgary, Calgary, AB, Canada; 7 Hotchkiss Brain Institute, Cumming School of Medicine, University of Calgary, Calgary, AB, Canada; University of Missouri Columbia, UNITED STATES

## Abstract

**Background:**

Antipsychotic medications are commonly prescribed to critically ill adult patients and initiation of new antipsychotic prescriptions in the intensive care unit (ICU) increases the proportion of patients discharged home on antipsychotics. Critically ill adult patients are also frequently exposed to multiple psychoactive medications during ICU admission and hospitalization including benzodiazepines and opioid medications which may increase the risk of psychoactive polypharmacy following hospital discharge. The associated impact on health resource utilization and risk of new benzodiazepine and opioid prescriptions is unknown.

**Research question:**

What is the burden of health resource utilization and odds of new prescriptions of benzodiazepines and opioids up to 1-year post-hospital discharge in critically ill patients with new antipsychotic prescriptions at hospital discharge?

**Study design & methods:**

We completed a multi-center, propensity-score matched retrospective cohort study of critically ill adult patients. The primary exposure was administration of ≥1 dose of an antipsychotic while the patient was admitted in the ICU and ward with continuation at hospital discharge and a filled outpatient prescription within 1-year following hospital discharge. The control group was defined as no doses of antipsychotics administered in the ICU and hospital ward and no filled outpatient prescriptions for antipsychotics within 1-year following hospital discharge. The primary outcome was health resource utilization (72-hour ICU readmission, 30-day hospital readmission, 30-day emergency room visitation, 30-day mortality). Secondary outcomes were administration of benzodiazepines and/or opioids in-hospital and following hospital discharge in patients receiving antipsychotics.

**Results:**

1,388 propensity-score matched patients were included who did and did not receive antipsychotics in ICU and survived to hospital discharge. New antipsychotic prescriptions were not associated with increased health resource utilization or 30-day mortality following hospital discharge. There was increased odds of new prescriptions of benzodiazepines (adjusted odds ratio [aOR] 1.61 [95%CI 1.19–2.19]) and opioids (aOR 1.82 [95%CI 1.38–2.40]) up to 1-year following hospital discharge in patients continuing antipsychotics at hospital discharge.

**Interpretation:**

New antipsychotic prescriptions at hospital discharge are significantly associated with additional prescriptions of benzodiazepines and opioids in-hospital and up to 1-year following hospital discharge.

## Introduction

Antipsychotic medication prescribing frequently occurs among critically ill patients to manage symptoms related to delirium, anxiety, or insomnia [1]. Several large randomized control studies have shown antipsychotic medication use in critically ill adult patients does not reduce incidence and duration of delirium, leading current guidelines to recommend against the routine use of antipsychotic medication for the prevention or treatment of delirium [2–6]. Antipsychotics are also commonly continued at hospital discharge. Previous observational studies estimate 21 to 24% of antipsychotic naïve critically ill patients who receive an antipsychotic in the intensive care unit (ICU) are discharged from hospital with an ongoing antipsychotic prescription [7,8]. Patients with critical illness are exposed to a number of psychoactive medications in addition to antipsychotics during their ICU admission and may be at risk of psychoactive polypharmacy (i.e., simultaneous use of at least two classes of psychoactive medications) following hospital discharge [9–11]. Benzodiazepine and opioid use in critical illness may contribute to negative clinical outcomes following critical illness including delirium, post-traumatic stress disorder, depression, anxiety, and cognitive dysfunction [12–14]. We utilized a multi-center, propensity-score matched retrospective cohort of critically ill patients receiving antipsychotic medications in the ICU to estimate health resource utilization outcomes and 30-day mortality following hospital discharge, and to evaluate the association between antipsychotic medication prescribing and the subsequent prescriptions of benzodiazepines and opioids in-hospital and up to 1-year post-hospital discharge.

## Methods

### Study design

This multi-center, propensity-score matched retrospective cohort study is reported according to the Strengthening the Reporting of Observational Studies in Epidemiology (STROBE) statement [15] (S1 File).

### Study setting & population

Adult patients at least 18 years of age admitted to one of four medical-surgical ICUs with electronic medical records in Calgary, Alberta, Canada (catchment population 1.5 million) from January 1, 2014 to June 30, 2016 were included in the study cohort. Patients were excluded if they had an antipsychotic medication prescription three months prior to hospital admission, length of ICU admission of <24 hours, ICU admission diagnosis of overdose, or if they died in the ICU or in hospital following ICU admission. Patients were further excluded if patient data did not link to a Discharge Abstract Database (DAD) admission or if they were non-Alberta residents. If patients had more than one admission to the ICU during the study period, only the first admission with a length of stay >24 hours was included. The primary study cohort was comprised of patients who survived to hospital discharge with follow-up on antipsychotic medication prescriptions up to 1-year following hospital discharge.

The included medical-surgical ICUs were closed units staffed by specialty accredited intensive care physicians that provide invasive mechanical ventilation, vasopressor medications, and invasive monitoring. Data for antipsychotic prescriptions were available from October 1, 2013 to June 30, 2017. Opioid and benzodiazepine prescriptions were available from January 1, 2014 to June 30, 2017.

### Data sources

We used data from seven databases as sources for this study, which have previously been utilized for research purposes [16–19]. eCritical Alberta is integrated within a bedside electronic medical record (MetaVision) that collects clinical and demographic data for ICU admitted patients in Alberta [17]. The DAD collects hospital outcome and diagnosis data on all hospital patients via diagnostic codes based on the International Classification of Disease, 10^th^ Revision, Canadian Enhancement [19]. Vital Statistics Database captures mortality data following ICU/hospital admission. The National Ambulatory Care Reporting System reports data for all emergency department visits. Sunrise Clinical Manager (SCM) is an electronic health record that captures computerized physician order entry and flowsheet documentation capturing ICU and hospital drug orders and patient drug administration [20]. The Pharmaceutical Information Network (PIN) is a web-enabled application within the Alberta Electronic Health Record that provides outpatient medication dispensing data and prescription information (i.e., quantity and compound elements) from community prescribers [21]. Data were linked deterministically via a unique provincial healthcare number. Authors did not have access to information that could identify participants during or after data collection. Included medications were identified and linked via Anatomical Therapeutic Chemical classifications.

### Exposure measures

The primary exposure was the administration of ≥1 dose of antipsychotic medication while the patient was admitted in the ICU. Included antipsychotic medications were those medications that have been previously reported in the literature to be commonly administered and based on clinical experience [7,8,22,23]. Antipsychotic medications included were haloperidol, quetiapine, risperidone, olanzapine, and methotrimeprazine with all formulations considered. All other antipsychotic medications were excluded as the listed antipsychotics represent the available formulary medications at included centres and are the most common clinically utilized antipsychotics reported in the critical care literature [7,8,11,22]. Antipsychotic medication continuation at hospital discharge was defined as those patients initially admitted to ICU who survived to hospital discharge, had administration of ≥1 dose of an antipsychotic medication in the ICU and hospital ward and additionally filled an outpatient prescription for an antipsychotic medication anytime within 1-year following hospital discharge.

### Outcome measures

The primary outcome was health resource utilization defined as 72-hour ICU readmission, 30-day hospital readmission, 30-day emergency room visitation, and 30-day mortality. ICU readmission was defined as readmission to ICU within 72-hours of transfer from ICU within the same hospitalization. Hospital readmission was defined as subsequent admission to hospital within 30-days of discharge from hospital for the index admission. Emergency room visitation was defined as subsequent visitation to the emergency room within 30-days following hospital discharge. Mortality was defined as death from any cause up to 30-days following hospital discharge.

Secondary outcomes included co-prescription of benzodiazepines and/or opioids during hospitalization (i.e., ICU or hospital ward) in addition to antipsychotic medication during hospitalization (i.e., ICU and hospital ward), and outpatient prescription of benzodiazepines and/or opioids up to 1-year post-hospital discharge. Prescription of a benzodiazepine and/or opioid medication in-hospital was defined as administration of ≥1 dose of any benzodiazepine and/or opioid medication during hospitalization either in the ICU or hospital ward. New outpatient prescription of psychoactive medications was defined as filling of a prescription for a pre-specified antipsychotic medication as an outpatient within 1-year following hospital discharge and an opioid or benzodiazepine medication within 1-year following hospital discharge. Included opioid drugs were hydromorphone, morphine, fentanyl, oxycodone, and codeine. Included benzodiazepine drugs were lorazepam, diazepam, temazepam, clonazepam, alprazolam, and triazolam.

### Covariate measures

Covariate measure were determined *a priori* based on clinical experience and previous studies [7,8,19,24,25]. Patient factors included age, sex, presence of each individual comorbidity from the Charlson Comorbidity Index (diabetes, chronic lung disease, chronic kidney disease, liver disease, cancer, chronic heart or peripheral vascular disease, neurological disease), illness severity upon ICU admission (SOFA score on admission), admission reason (medical, surgical, neurological, trauma), use of invasive mechanical ventilation, use of vasoactive medications, use of continuous renal replacement therapy, opioid use in ICU, benzodiazepine use in ICU, and ICU length of stay category (<3, 3 to <7, ≥7 days).

### Statistical analysis

A propensity-score matched cohort of patients who received or did not receive an antipsychotic medication during hospitalization was created. Propensity-scores were based on age, sex, admission reason (medical, surgical, neurological, trauma), presence/absence of each individual Charlson comorbidity (diabetes, chronic lung disease, chronic kidney disease, liver disease, cancer, chronic heart or peripheral vascular disease, neurological disease) [26], admission APACHE II score, use of invasive mechanical ventilation (yes/no), use of non-invasive mechanical ventilation (yes/no), use of vasoactive medications (yes/no), use of continuous renal replacement therapy (yes/no), use of opioids (yes/no), use of benzodiazepines (yes/no) and ICU length of stay category (<3, 3 to <7, ≥7 days). The cohort was based on 1:1 nearest neighbor matching without replacement using the logit of the propensity-score and specified caliper width equal to 0.05 of the standard deviation of the logit of the propensity-score.

Patient characteristics were summarized using median with interquartile range (IQR) and frequency with percent, as appropriate. Primary outcomes were analyzed using mixed effects logistic regression accounting for clustering of patients within ICU sites. Among the overall cohort, mixed effects logistic regression models were adjusted for the same variables that were included in the propensity-score. Secondary outcomes were also analyzed using mixed effects logistic regression and adjusted for the same variables included in the propensity-score. A two-sided p-value of <0.05 was used to define statistical significance. Analyses were completed using R (version 4.0.0). Propensity-score matching was completed using the R package “MatchIt” (version 3.0.2) and mixed effects logistic regression models were done using the R package “lme4” (version 1.1–23) [27].

### Ethics approval

This study was approved by the University of Calgary Conjoint Health Research Ethics Board (REB17-0389). Ethics approval allowed for database linkage and waiver of individual patient consent.

## Results

### Study population

**Fig 1:**
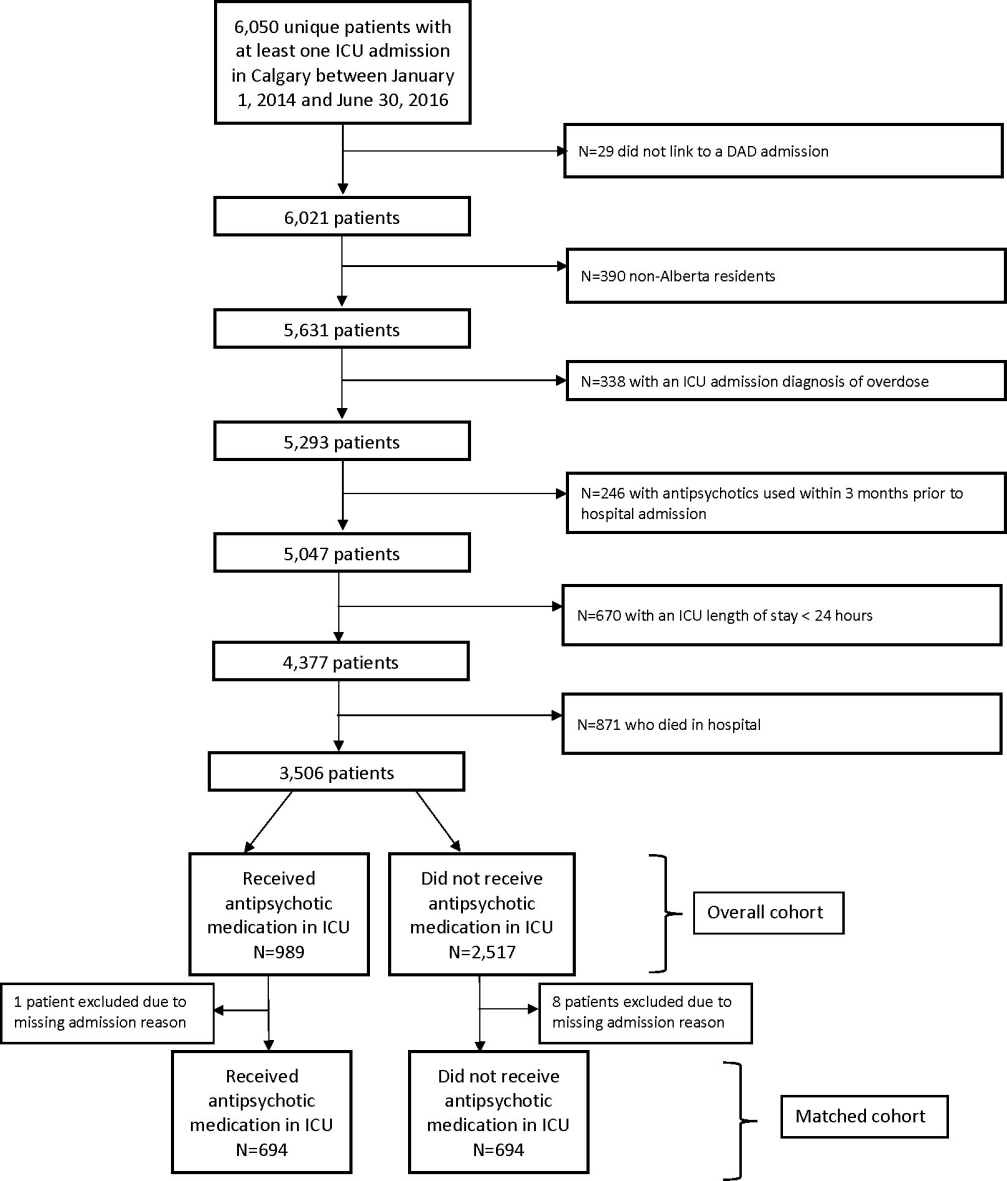
Study cohort flow diagram.

There were 6,050 eligible patients admitted to one of four medical-surgical ICUs with at least one ICU admission between January 1, 2014 and June 30, 2016. After exclusion of ineligible patients, the overall study cohort for analysis consisted of 3,506 patients who survived to hospital discharge. (Fig 1) Due to missing admission reason for one patient who received antipsychotic medications and eight patients who did not receive antipsychotic medications from the overall cohort, propensity-scores were calculated for 988 patients who received antipsychotic medications in the ICU (99.9%) and 2,509 patients who did not receive antipsychotic medications in the ICU (99.7%). Of those patients with complete data, 294 patients who received antipsychotic medications in ICU and 1,815 patients who did not receive antipsychotic medications in ICU could not be matched with outcome data within the specified caliper width equal to 0.05. This resulted in 1,388 patients included in the matched cohort (Table 1). An additional 160 patients were excluded from the secondary analysis as they only received an antipsychotic medication on the hospital ward (and not in ICU). Baseline patient and hospital level characteristics of all patients admitted to the ICU during this study period can be found in S1 Table.

**Table 1:** Patient and hospital characteristics of patients who survived hospital stay.

	Overall Cohort (n = 3,506)		Matched Cohort (n = 1,388)	
	Received antipsychotic medication in ICU N = 989	Did not receive antipsychotic medication in ICU N = 2,517	Received antipsychotic medication in ICU N = 694	Did not receive antipsychotic medication in ICU N = 694
**Patient characteristics on Admission to ICU**				
Female	326 (33.0)	1077 (42.8)	248 (35.7)	272 (39.2)
Age, median [IQR]	60 (47–70)	58 (44–69)	60 (48–71)	60 (49–69)
**Comorbidities**				
Diabetes	258 (26.1)	620 (24.6)	189 (27.2)	181 (26.1)
Chronic lung disease	165 (16.7)	339 (13.5)	105 (15.1)	115 (16.6)
Renal	43 (4.3)	115 (4.6)	33 (4.8)	36 (5.2)
Liver disease	82 (8.3)	133 (5.3)	43 (6.2)	44 (6.3)
Malignancy	83 (8.4)	312 (12.4)	64 (9.2)	61 (8.8)
Chronic heart or peripheral vascular disease	210 (21.2)	442 (17.6)	148 (21.3)	152 (21.9)
Neurological disease^a^	111 (11.2)	235 (9.3)	87 (12.5)	78 (11.2)
Any comorbidity	661 (66.8)	1583 (62.9)	468 (67.4)	460 (66.3)
**Admission type** ^**b**^				
Elective surgery	38 (3.8)	208 (8.3)	31 (4.5)	31 (4.5)
Emergent surgery	176 (17.8)	520 (20.7)	124 (17.9)	150 (21.6)
No surgery	774 (78.3)	1782 (71.0)	539 (77.7)	513 (73.9)
**Admission reason** ^**c**^				
Medical	587 (59.4)	1405 (56.0)	405 (58.4)	416 (59.9)
Surgical	180 (18.2)	637 (25.4)	133 (19.2)	139 (20.0)
Neurological	88 (8.9)	262 (10.4)	74 (10.7)	67 (9.7)
Trauma	133 (13.5)	205 (8.2)	82 (11.8)	72 (10.4)
**Location before admission**				
Emergency department	505 (51.1)	1200 (47.7)	350 (50.4)	337 (48.6)
Inter-facility	21 (2.1)	25 (1.0)	11 (1.6)	7 (1.0)
Operating room	162 (16.4)	599 (23.8)	119 (17.1)	145 (20.9)
Other	24 (2.4)	58 (2.3)	16 (2.3)	22 (3.2)
Ward	277 (28.0)	635 (25.2)	198 (28.5)	183 (26.4)
SOFA score, median [IQR]	7 (5–10)	5 (3–8)	7 (5–9)	7 (4–9)
APACHE II score, median [IQR]	21 (16–26)	17 (12–22)	20 (15–25)	20 (14–25)
**Interventions received in ICU**				
Invasive mechanical ventilation received	872 (88.2)	1651 (65.6)	581 (83.7)	578 (83.3)
Non-invasive mechanical ventilation received	173 (17.5)	321 (12.8)	116 (16.7)	123 (17.7)
Vasoactive medications received	648 (65.5)	1060 (42.1)	411 (59.2)	411 (59.2)
Renal replacement therapy	90 (9.1)	79 (3.1)	52 (7.5)	43 (6.2)
**Patient characteristics on ICU discharge**				
SOFA score at discharge, median (IQR)	1 (0–3)	0 (0–3)	1 (0–3)	0 (0–3)
ICU length of stay (days), median [IQR]	10.0 (6.0–16.0)	3.7 (2.2–6.3)	7.8 (4.9–13.2)	7.4 (4.2–10.5)
**Patient characteristics on hospital discharge**				
Hospital length of stay (days), median [IQR]	26.7 (15.4–52.2)	13.5 (7.0–27.8)	24.3 (13.6–51.2)	20.5 (10.7–46.1)
**Hospital Characteristics**				
Teaching hospital	855 (86.5)	2176 (86.5)	606 (87.3)	610 (87.9)
≥ 600 hospital beds	615 (62.2)	1497 (59.5)	439 (63.3)	387 (55.8)
≥ 20 ICU beds	430 (43.5)	1106 (43.9)	305 (43.9)	298 (42.9)
ICU occupancy at discharge ≥ 80%	772 (78.1)	1939 (77.0)	536 (77.2)	538 (77.5)
**Antipsychotic medication used in ICU** ^**e**^				
Quetiapine	807 (81.6)	N/A	542 (78.1)	N/A
Haloperidol	431 (43.6)	N/A	291 (41.9)	N/A
Olanzapine	176 (17.8)	N/A	109 (15.7)	N/A
Risperidone	7 (0.7)	N/A	5 (0.7)	N/A
Methotrimeprazine	8 (0.8)	N/A	2 (0.3)	N/A
Opioid medication administration^d^	843 (85.2)	1555 (61.8)	555 (80.0)	558 (80.4)
Benzodiazepine medication administration^d^	740 (74.8)	803 (31.9)	450 (64.8)	460 (66.3)
**Antipsychotic continuation at hospital discharge among those who went from ICU to ward**	N = 895 patients discharged home from ward	N = 2144 patients discharged home from ward	N = 628 patients discharged home from ward	N = 599 patients discharged home from ward
**Any antipsychotic continuation**	207 (20.9)	N/A	132 (21.0)	N/A
Quetiapine	149 (15.1)	N/A	91 (14.5)	N/A
Haloperidol	25 (2.5)	N/A	20 (3.2)	N/A
Olanzapine	46 (4.7)	N/A	32 (5.1)	N/A
Risperidone	23 (2.3)	N/A	18 (2.9)	N/A
Methotrimeprazine	0 (0.0)	N/A	0 (0.0)	N/A

^a^ As defined by the Charlson Comorbidity Index.

^b^ Missing for 1 patient who received antipsychotics and 7 patients who did not receive antipsychotics.

^c^ Missing for 1 patient who received antipsychotics and 8 patients who did not receive antipsychotics.

^d^ Defined as at least one dose administration while receiving antipsychotic medication.

^e^ Percentages do not add up to 100 due to the possibility of multiple antipsychotics prescribed per patient.

Baseline characteristics were balanced in the propensity-score matched cohort (Table 1). Most patients in the matched cohort were admitted via the emergency department for medical admission reasons. The median APACHE II score of those who received antipsychotic medications in the ICU was 20 [IQR 15–25] and those who did not receive antipsychotic medications was 20 [IQR 14–25]. The median age of patients in the propensity-score matched cohort in both patients who received antipsychotic medications and those who did not was 60 years [IQR 48–71], [IQR 49–69]. Median ICU length of stay was similar between those patients who did receive antipsychotic medications in ICU (7.8 days [IQR 4.9–13.2]) and those who did not receive antipsychotic medications in ICU (7.4 days [IQR 4.2–10.5]). Median length of hospital stay was similar for those patients who did receive antipsychotic medications in ICU (24.3 days [IQR 13.6–51.2]) compared to those patients who did not receive antipsychotic medications in ICU (20.5 days [IQR 10.7–46.1]).

### Medication administration outcomes

The proportion of ICU patients in the overall cohort of those patients that survived their ICU and hospital stay and received at least one dose of antipsychotic medication during their ICU admission was 989 (28.2%). Among those who received antipsychotics in the matched cohort, quetiapine (542 (78.1%)), and haloperidol (291 (41.9%)) were the most common prescribed antipsychotics to patients in the ICU. Some patients prescribed antipsychotics were prescribed multiple antipsychotic types (222/694 (32.0%)). Of the ICU patients that received an antipsychotic medication in the ICU and survived to hospital discharge, 132 (21.0%) patients were prescribed an antipsychotic medication at hospital discharge. The most common antipsychotic medications continued at hospital discharge were quetiapine (91 (14.5%)) and olanzapine (32 (5.1%)) (Table 1).

**Fig 2:**
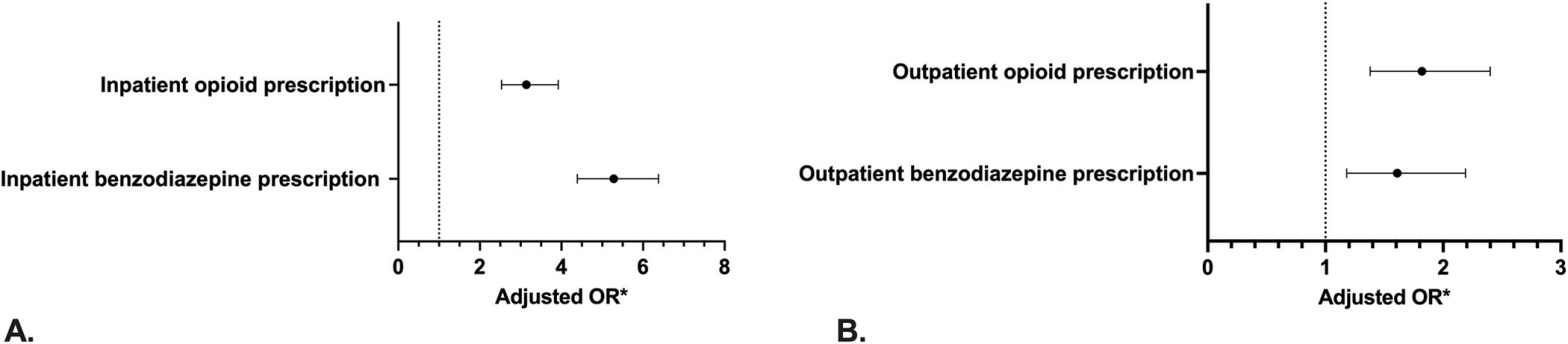
Mixed effects logistic regression analysis of co-prescription of opioids or benzodiazepines in (A) hospitalized patients^a^ and (B) outpatients surviving hospital who received antipsychotic medications during ICU and on hospital ward compared to those who did not receive antipsychotic medications. *Adjusted for sex, age, presence/absence of each of the individual Charlson comorbidities (diabetes, chronic lung disease, chronic kidney disease, liver disease, cancer, chronic heart or peripheral vascular disease, neurological disease), APACHE I score on admission, admission reason (medical, surgical, neurological, trauma), invasive ventilation, non-invasive ventilation, vasoactive medications, continuous renal replacement therapy. ^a^For those who received antipsychotics, this includes patients who received both antipsychotic and benzodiazepine or opioid in ICU or patients who received both antipsychotic and benzodiazepine or opioid in hospital.

In ICU patients that received an ongoing antipsychotic medication during their hospitalization, there was an increased odds of the co-prescription of benzodiazepines (adjusted OR 5.29 [95%CI 4.38–6.39]) as well as the co-prescription of opioids (adjusted OR 3.18 [95%CI 2.55–3.96]) during their hospitalization compared to those who did not receive antipsychotic medications during their hospitalization. (Fig 2A) Of the ICU patients that received an antipsychotic medication in the ICU that was prescribed following hospital discharge, there was an increased odds of patients receiving new concurrent prescriptions for a benzodiazepine medication (adjusted OR 1.61 [95%CI 1.19–2.19]) or an opioid medication (adjusted OR 1.82 [95%CI 1.38–2.40]) up to one-year following hospital discharge (Fig 2B).

### Health resource utilization outcomes

There was no association between ICU patients that received an antipsychotic in the ICU or the ward and 72-hour ICU readmission and 30-day emergency room visitation (Table 2). There was an increased risk of 30-day hospital readmission in the overall cohort of ICU patients that received antipsychotic medication in the ICU or ward compared to ICU patients who did not receive antipsychotic medications in the ICU or ward (adjusted OR 1.28 [95%CI 1.04–1.59). This relationship was not significant in the propensity-score matched cohort (adjusted OR 1.18 [95%CI 0.92–1.52]).

**Table 2:** Mixed effects logistic regression analysis of antipsychotic use and health resource utilization following hospital discharge among those who survived hospital stay.

	Overall Cohort (n = 3,506)			Propensity score-matched cohort (n = 1,388)^b^		
Outcomes	Received antipsychotic medication N = 989	Did not receive antipsychotic medication N = 2,517	Adjusted Odds Ratio (95% CI)^a^	Received antipsychotic medication N = 694	Did not receive antipsychotic medication N = 694	Odds Ratio (95% CI)
72h ICU readmission	20 (2.0)	49 (1.9)	0.96 (0.50–1.80)	10 (1.4)	12 (1.7)	0.83 (0.35–1.94)
30d hospital readmission	242 (24.5)	457 (18.2)	1.28 (1.04–1.59)	173 (24.9)	152 (21.9)	1.18 (0.92–1.52)
30d emergency room visitation	202 (20.4)	531 (21.1)	1.00 (0.80–1.24)	141 (20.3)	148 (21.3)	0.94 (0.73–1.22)
30d mortality	18 (1.8)	44(1.7)	0.89 (0.45–1.71)	14 (2.0)	11 (1.6)	1.28 (0.58–2.90)

^a^ Adjusted for sex, age, presence/absence of each of the individual Charlson comorbidities (diabetes, chronic lung disease, chronic kidney disease, liver disease, cancer, chronic heart or peripheral vascular disease, neurological disease), SOFA score on admission, admission reason (medical, surgical, neurological, trauma), invasive ventilation, non-invasive ventilation, vasoactive medications, continuous renal replacement therapy, opioid use in ICU, benzodiazepine use in ICU and ICU length of stay category (<3, 3 to <7, ≥7 days).

^b^ Propensity scores were based on age, sex, admission reason (medical, surgical, neurological, trauma), presence/absence of each of the individual Charlson comorbidities (diabetes, chronic lung disease, chronic kidney disease, liver disease, cancer, chronic heart or peripheral vascular disease, neurological disease), admission APACHE II score, use of invasive mechanical ventilation (yes/no), use of non-invasive mechanical ventilation (yes/no), use of vasoactive medications (yes/no), use of continuous renal replacement therapy (yes/no), use of opioids (yes/no), use of benzodiazepines (yes/no) and ICU length of stay category (<3, 3 to <7, ≥7 days). The cohort was based on 1:1 nearest neighbor matching without replacement using the logit of the propensity score and specified caliper width equal to 0.05 of the standard deviation of the logit of the propensity score.

### Mortality outcomes

There was no association between 30-day mortality following hospital discharge in those ICU patients who received an antipsychotic medication in ICU continued to hospital discharge compared to those ICU patients who did not receive an antipsychotic medication in ICU (adjusted OR 1.28 [95%CI 0.58–2.84]) (Table 2).

## Discussion

In this multi-center propensity-matched retrospective study, we identified that one in five antipsychotic naïve ICU patients who received an antipsychotic medication in ICU were subsequently prescribed an antipsychotic medication at hospital discharge. Being prescribed an antipsychotic medication during hospitalization was associated with both an increased odds of being co-prescribed a benzodiazepine and opioid medication during hospitalization and in the 1-year following a patient’s hospital discharge.

Our results confirm and expand on previous literature describing the prescribing practices of antipsychotics during ICU admission and following hospital discharge in patients who experience critical illness [7,8,22,28,29]. Similar to previously reported results, our study found that quetiapine and olanzapine were the most common antipsychotic medications continued at hospital discharge in this ICU patient cohort [7,8]. This prescribing practice may reflect a trend toward the more common use of atypical antipsychotics for their sedating histaminergic effects for sleep and restlessness following resolution of delirium in an attempt to avoid prescribing benzodiazepines [30–32]. Additionally, benzodiazepines may be co-prescribed with opioids to help manage symptoms related to chronic pain and anxiety experienced following critical illness. A recent publication from Olafson *et al*. [33] examined the long-term use of ongoing psychotropic medications in a 5-year pre- and post-hospitalization period of 49,439 ICU patients compared to non-ICU hospitalized patients and identified an increased association of psychotropic medication use in the five years after ICU admission. A higher proportion of ICU patients in this cohort not only received antipsychotic medications but also antidepressants, anxiolytics, and sedative-hypnotics compared to their control group of non-ICU patients [33]. In contrast to our findings, the ICU patients in this cohort had a lower prevalence of long-term antipsychotic medication use following hospital discharge. This may be an underestimation of the association as their comparator group of non-ICU hospitalizations had a higher proportion of patients with underlying mental health disorders potentially requiring ongoing antipsychotic medications and may explain the observed differences.

To the best of our knowledge, our study is the first study to evaluate the independent association of continued antipsychotic prescribing in critically ill patients to hospital discharge on health resource utilization outcomes. We did not find an association between 72-hour ICU readmission, 30-day hospital readmission, 30-day emergency room visitation, and 30-day mortality in those ICU patients who received an antipsychotic medication in ICU continued to hospital discharge compared to those ICU patients who did not receive an antipsychotic medication in ICU. Our findings suggest that despite an increased association between ongoing antipsychotic medication use and benzodiazepine and opioid medication prescriptions, this does not translate into increased short-term healthcare resource utilization costs. However, these results do not account for potential increased ambulatory outpatient utilization which is known to occur in critically ill patients post-hospital discharge [34–36].

Our findings identify a novel and concerning association between antipsychotic medication use following hospital discharge in patients who experience critical illness and subsequent benzodiazepine and opioid prescription in the 1-year following hospital discharge potentially reflecting an ICU patient population at risk of long-term psychoactive polypharmacy following hospital discharge [10,11,37]. The question remains whether this increased risk of psychoactive medication prescription in the 1-year following hospital discharge further represents an association with poor patient functional and quality of life outcomes [38,39]. It is possible that patients who receive antipsychotic medications during their ICU admission may be at higher risk of frailty following hospital discharge and likewise may have worse physical and cognitive health related quality of life and greater functional dependence leading to psychoactive medication use [38–40]. Further evaluation of the association between this patient population and patient-centered functional outcomes are needed to better understand the contribution of frailty and chronic pain to these findings. This patient population may represent individuals particularly at risk of polypharmacy that may benefit from outpatient medication reconciliation by clinician experts in the post-ICU setting [38,39,41,42].

## Strengths and limitations

Our retrospective study has several strengths including the large sample size with complete data on pre- and post-hospital medication prescribing up to 3-months prior to hospitalization and 1-year following hospital discharge. The use of propensity-score matched analysis controls for many patient-level and hospital-level confounders is also a strength. Previous evaluations of common demographic and clinical variables suggest good agreement between documentation in the medical record and the integrated critical care electronic medical record system, TRACER, used for data collection [43]. Despite using propensity-score matched analysis, residual confounding remains possible for factors associated with the risk of being prescribed a benzodiazepine or opioid medication that were not included as a variable such as new psychiatric diagnoses acquired because of ICU admission. Our study also has limitations. We aimed to exclude patients with appropriate clinical indications for antipsychotic medication prescriptions by excluding those patients with pre-hospital antipsychotic medication prescriptions 3-months prior to hospitalization and those patients admitted for drug overdoses as a surrogate of possible underlying mental health disorders. It is possible that evaluating antipsychotic prescriptions 3-months prior to hospitalization may not be a sufficient timeframe to exclude all patients that utilize antipsychotics for psychiatric illness clinical indications. We were unable to exclude all patients admitted for a primary psychiatric reason nor determine the proportion of patients that were appropriately prescribed antipsychotics, benzodiazepines, or opioids. Although this would potentially overrepresent the relationship between antipsychotic and benzodiazepine or opioid prescribing, the proportion of patients admitted for a primary psychiatric diagnosis missed by our exclusion criteria is expected to be small. Future studies may consider excluding patients with previous inpatient psychiatric hospitalizations or psychiatric illness diagnosis codes within their medical record as an additional strategy to identify patients that may be admitted to the ICU for a primary psychiatric diagnosis. Additionally, it was not possible to accurately discern the clinical indication for the prescription of antipsychotics, opioids, and benzodiazepines in-hospital or in the outpatient setting. Thus, there may be multifactorial indications for which these medications are being prescribed to patients during and following hospitalization. This remains an important area of future investigation to better guide clinical experts completing outpatient medication reconciliation. Our results are generalizable to an ICU patient population receiving care in a universal healthcare system with established hospital electronic medical record systems and accessible ambulatory care pathways. Generalizability may be limited in private or mixed public and private healthcare systems as well as those healthcare systems without established mechanisms for continuity of care from hospital discharge to the outpatient environment.

## Conclusion

Among ICU patients surviving to hospital discharge who continue new antipsychotic medication prescriptions at hospital discharge, there was an association with new additional prescriptions of benzodiazepines and opioids in-hospital and up to 1-year following hospital discharge. There was no association between health resource utilization or 30-day mortality following hospital discharge and ongoing antipsychotic medication use at hospital discharge in this patient cohort. This ICU patient population may benefit from additional medication reconciliation prior to or following hospital discharge.

## Supporting information

S1 Table Patient and hospital characteristics among all patients admitted to ICU during study period. (DOCX)

S1 File STROBE Statement for cohort studies. (DOCX)
