# Supplementary material for: Impacts of antipsychotic medication prescribing practices in critically ill adult patients on health resource utilization and new psychoactive medication prescriptions
Source: PLoS One. 2023 Jun 29;18(6):e0287929. doi: 10.1371/journal.pone.0287929 (PMC10310007; doi:10.1371/journal.pone.0287929)
Supplement: S1 Table — (DOCX) [file pone.0287929.s001.docx]

**S1 Table.** Patient and hospital characteristics among all patients admitted to ICU during study period

|  | Overall  N = 4,377 | Received antipsychotic medication in ICU  N = 1,203 | Did not receive antipsychotic medication in ICU  N = 3,174 |
| --- | --- | --- | --- |
| **Patient characteristics on Admission to ICU** |  |  |  |
| Female | 1,739 (39.7) | 404 (33.6) | 1,335 (42.1) |
| Age, median [IQR] | 60 (47-71) | 62 (50-71) | 60 (46-71) |
| **Comorbidities** |  |  |  |
| Diabetes | 1,130 (25.8) | 329 (27.3) | 801 (25.2) |
| Chronic lung disease | 628 (14.3) | 215 (17.9) | 413 (13.0) |
| Renal | 213 (4.9) | 55 (4.6) | 158 (5.0) |
| Liver disease | 337 (7.7) | 114 (9.5) | 223 (7.0) |
| Malignancy | 543 (12.4) | 123 (10.2) | 420 (13.2) |
| Chronic heart or peripheral vascular disease | 879 (20.1) | 284 (23.6) | 595 (18.7) |
| Neurological disease^a^ | 498 (11.4) | 137 (11.4) | 361 (11.4) |
| Any comorbidity | 2,943 (67.2) | 846 (70.3) | 2,097 (66.1) |
| **Admission type^b^** |  |  |  |
| Elective surgery | 270 (6.2) | 41 (3.4) | 229 (7.2) |
| Emergent surgery | 857 (19.6) | 218 (18.1) | 639 (20.2) |
| No surgery | 3,241 (74.2) | 943 (78.5) | 2,298 (72.6) |
| **Admission reason^c^** |  |  |  |
| Medical | 2,509 (57.5) | 736 (61.2) | 1,773 (56.0) |
| Surgical | 976 (22.3) | 228 (19.0) | 748 (23.6) |
| Neurological | 483 (11.1) | 99 (8.2) | 384 (12.1) |
| Trauma | 399 (9.1) | 139 (11.6) | 260 (8.2) |
| **Location before admission** |  |  |  |
| Emergency department | 2,110 (48.2) | 593 (49.3) | 1,517 (47.8) |
| Inter-facility | 54 (1.2) | 24 (2.0) | 30 (0.9) |
| Operating room | 901 (20.6) | 196 (16.3) | 705 (22.2) |
| Other | 99 (2.3) | 27 (2.2) | 72 (2.3) |
| Ward | 1,213 (27.7) | 363 (30.2) | 850 (26.8) |
| SOFA score, median [IQR] | 6 (4-9) | 8 (5-10) | 6 (4-9) |
| APACHE II score, median [IQR] | 19 (14-25)) | 22 (17-27) | 18 (13-25) |
| **Interventions received in ICU** |  |  |  |
| Invasive mechanical ventilation received | 3,293 (75.2) | 1,060 (88.1) | 2,233 (70.4) |
| Non-invasive mechanical ventilation received | 617 (14.1) | 236 (19.6) | 381 (12.0) |
| Vasoactive medications received | 2,381 (54.4) | 832 (69.2) | 1,549 (48.8) |
| Renal replacement therapy | 295 (6.7) | 130 (10.8) | 165 (5.2) |
| **Patient characteristics on ICU discharge** |  |  |  |
| SOFA score at discharge, median (IQR) | 1 (0-4) | 1 (0-4) | 1 (0-4) |
| ICU length of stay (days), median [IQR] | 4.9 (2.7-9.4) | 10.0 (6.1-16.2) | 3.7 (2.2-6.6) |
| **Patient characteristics on hospital discharge** |  |  |  |
| Hospital length of stay (days), median [IQR] | 15.3 (7.4-31.9) | 24.6 (14.6-48.6) | 11.9 (6.0-25.3) |
| **Hospital Characteristics** |  |  |  |
| Teaching hospital | 3,811 (87.1) | 1,036 (86.1) | 2,775 (87.4) |
| ≥ 600 hospital beds | 2,661 (60.8) | 737 (61.3) | 1,924 (60.6) |
| ≥ 20 ICU beds | 1,959 (44.8) | 501 (41.6) | 1,458 (45.9) |
| ICU occupancy at discharge ≥ 80% | 3,376 (77.1) | 914 (76.0) | 2,462 (77.6) |
| **Antipsychotic medication used in ICU** |  |  |  |
| Quetiapine | 955 (21.8) | 955 (79.4) | N/A |
| Haloperidol | 540 (12.3) | 540 (44.9) | N/A |
| Olanzapine | 218 (5.0) | 218 (18.1) | N/A |
| Risperidone | 9 (0.2) | 9 (0.7) | N/A |
| Methotrimeprazine | 12 (0.3) | 12 (1.0) | N/A |
| Opioid medication administration^b^ | 3,113 (71.1) | 1,036 (86.1) | 2,077 (65.4) |
| Benzodiazepine medication administration^b^ | 2,065 (47.2) | 903 (75.1) | 1,162 (36.6) |
| **Antipsychotic continuation at transfer to ward among those who survived ICU and went to ward** | N=3,269 patients transferred alive from ICU to ward | N=972 patients transferred alive from ICU to ward | N=2,297 patients transferred alive from ICU to ward |
| **Any antipsychotic continuation** | N/A | 529 (54.4) | N/A |
| Quetiapine | N/A | 428 (44.0) | N/A |
| Haloperidol | N/A | 113 (11.6) | N/A |
| Olanzapine | N/A | 92 (9.5) | N/A |
| Risperidone | N/A | 20 (2.1) | N/A |
| Methotrimeprazine | N/A | 12 (1.2) | N/A |
| **Antipsychotic continuation at hospital discharge among those who went from ICU to ward and survived hospital** | N=3,039 patients surviving hospital and discharged home from ward | N=895 patients surviving hospital and discharged home from ward | N=2,144 patients surviving hospital and discharged home from ward |
| **Any antipsychotic continuation** | N/A | 199 (22.2) | N/A |
| Quetiapine | N/A | 144 (16.1) | N/A |
| Haloperidol | N/A | 24 (2.7) | N/A |
| Olanzapine | N/A | 43 (4.8) | N/A |
| Risperidone | N/A | 23 (2.6) | N/A |
| Methotrimeprazine | N/A | 0 (0.0) | N/A |

^a^ As defined by the Charlson Comorbidity Index

^b^ Missing for 1 patient who received antipsychotics and 8 patients who did not receive antipsychotics

^c^ Missing for 1 patient who received antipsychotics and 9 patients who did not receive antipsychotics

^d^ Defined as at least one dose administration while receiving antipsychotic medication
